# Heavenly Messengers

**Published:** 1888-04

**Authors:** 


### HEAVENLY MESSENGERS.

At the last weekly meeting of the St. Vincent de Paul Conference, attached to the Church of St. Charles Borromeo, Brooklyn, an incident of strange interest was related by Mr. Charles A. Hoyt, a gentleman well known in the City of Churches by his deeds of charity and many kindly ways. He attends the meetings of this conference regularly, and usually relates some story of an edifying or instructive nature.

On this occasion the address of Mr. Hoyt was a history of a remarkable and miraculous case. It appears, according to his correspondent, that Rev. Jacob A. Walter, pastor of St. Patrick's Church, Washington, D. C., was seated in his study one evening, a short time ago, when a gentle knock at the door roused him from the duties in which he had been engaged. "Come in," said Father Walter; but no response was made. Fancying that he had made a mistake, he resumed his work only to be again distracted by the timorous knock. "Come in," he said again; but the door did not open, nor was he answered. He never lifted his head from his work, expecting that whoever it was would make his or her presence felt, when close to the table where he was seated. But again the knock came, the third time, and the good priest started for the door.

Father Walter is a practical man, not in the least imaginative, and he knew that there must be some one at the door. He opened it and was surprised to see two little girls standing right before him. "What is it you want, children?" asked the priest, quite forgetting to inquire how they came into the house without their progress being barred by the party whose duty it was to attend to the door. They answered that their father was dying, that he needed the last Sacraments and that the presence of Father Walter was required. They told him the building wherein the sick man lay, and, in his haste to reach him, Father Walter forgot to ask the name.

He made his way to the building and found the dying man on a mattress on the floor, with no friends near to smooth by their presence his passage to the grave. After attending to the spiritual wants of the man the good priest began to make inquiries about his temporal affairs.

"Why did you not send around to me earlier?" asked Father Walter.

"Because I am alone and had no one to send. I am glad you came this way."

"But," said Father Walter, "two children, saying you were their father, came to me."

"My two children are in heaven," was the reply. Father Walter described the children and the dying man recognized the description as that of his girls in life. He then understood the remarkable favor shown him and exclaimed: "They were my angel children, thanks be to the Lord."

The dying man, whose name was not mentioned by Mr. Hoyt, ascribed the grace with which his last moments were blessed by God to his regular practice of praying for the dead, and applying to the souls in Purgatory many of the indulgences he was privileged to earn. He believed that his prayers had been returned by the souls he had benefited and he departed this life calmly, trusting faithfully in the mercy of God.—*New York Catholic News*, March 4.

*Editor*, HALL'S JOURNAL OF HEALTH:

Progress is the modern gospel. An enlightened peace seems about to dawn upon the world. Where base contentions once prevailed, heavenly reciprocities at last shall stand forth shining with noonday splendor. Perhaps in no other direction does the rounding of old antagonism into beautiful symmetry appear to better advantage than in the spirit of the wide awake physician who has the spirit of our American civilization in him, a potent power. I refer to the death blow to quackery and maudling swindlers by the application of the test of advanced scientific information to the method of applying magnetic power, the scholarly healer, starting from the solid terra-firma, anatomy and physiology, carries his knowledge onward into biology, psychology and psychometry. Here he enters the sphere of causation. Physical science resurrected becomes a Savior. The process of induction in this higher realm, gives the anatomy of a nerve; and lays down with an authoritative (because demonstrable) voice the laws which govern the distribution of the nervous force of our organic being. Understanding these important data, the operator can direct his power as scientifically as the navigator sails the seas. This enlightened scientific data is the lamp to guide the potential curative wave of magnetic life. Nature in her grand processes, is refining matter, and with this process the law of organic chemistry continually acts with increased perfection. In humanity the phenomenon of progress in this direction is pleasant to behold. Our bodies are of a finer texture to-day than were the bodies of the generations that have preceded us. Hygienic information strictly adhered to causes the form of clay to become more refined. How essential then, that the magnetic operator should have a reservoir of power in a healthy organism, the direct correspondence of magnetism with the nervous system, gives it a marked superiority over all such grosser agents as must reach that delicate framework of life by a circuitious route. Of all remedies, this alone pours its benefits direct upon the very springs of sensation. Thus we possess a subtle means of acting efficiently upon that fountain head of calamity and disease, to which neither drug nor couching needle can find its way. Then All Hail! the true physician who is endowed with power scientifically imparting Magnetic Vital Force to the sick, and directing it wisely to the weak and diseased organs, stimulating them with new life and assisting Nature in her grand process of healing the *sick* and *infirm*. Thousands of so-called incurables have been restored to health and happiness by this method alone.

DUMONT C. DAKE, M. D.
304 Fifth Avenue, New York City.

